# “Everything else comes first”: a mixed-methods analysis of barriers to health behaviors among military spouses

**DOI:** 10.1186/s12889-018-5938-z

**Published:** 2018-08-15

**Authors:** Emily L. Mailey, Carrie Mershon, Jillian Joyce, Brandon C. Irwin

**Affiliations:** 1 Department of Kinesiology, Kansas State University, 8 Natatorium, Manhattan, KS 66506 USA; 2 Department of Food, Nutrition, Dietetics and Health, Kansas State University, 213 Justin Hall, Manhattan, KS 66506 USA

**Keywords:** Military spouses, Army, Barriers, Physical activity, Diet, Stress, Social support

## Abstract

**Background:**

Military spouses are integral to the health of their families, but have demonstrated elevated levels of stress, depression, and anxiety. Participating in health behaviors such as physical activity and healthy eating may have a positive impact on spouses’ physical and mental health, but emerging evidence suggests spouses’ participation in these behaviors is scarce. Thus, the purpose of this study was to examine the most frequently reported barriers to health behaviors among military spouses.

**Methods:**

Military spouses were recruited to complete surveys (*N* = 230) or participate in focus group sessions (*N* = 22). On the surveys, participants indicated up to 3 of their most frequent barriers to physical activity, diet, social connection, and stress management. Responses were coded and summed to identify the most commonly reported barriers to each health behavior. Subsequently, focus group sessions were conducted to gain a more in-depth understanding of the challenges military spouses face when trying to maintain a healthy lifestyle. Focus group transcripts were coded using thematic data analysis to identify the most frequently discussed barriers for each behavior.

**Results:**

On the surveys, lack of time was the most prevalent barrier for physical activity, social connection, and stress management, and the second most prevalent barrier for diet. Financial concerns were the most prevalent barrier to maintaining a healthy diet. Barriers related to parent/family responsibilities were commonly reported across all health behaviors. During the focus group sessions, the transient military lifestyle was reported to have a significant impact on all of the health behaviors. Other military-related stressors including deployments and the necessity to “do it all” alone were frequently discussed. Many participants exhibited rigid definitions of what “counts” as exercise or health eating. Overall, participants reported sacrificing participation in health behaviors to attend to other priorities.

**Conclusions:**

Military spouses reported numerous barriers to health behaviors that made it difficult for them to prioritize their own health and well-being. Although some of the barriers reported were similar to barriers reported by civilians, unique stressors associated with military life further impeded participation in health behaviors. These findings can be used to inform future health promotion interventions for military spouses.

## Background

Increasingly, military spouses are being recognized as a cornerstone of the health and wellness of the military family [1]. Research has shown soldiers with dissatisfied spouses are more likely to leave the military [2], and that family leadership by the spouse is critical to family wellness, particularly during deployment [3]. Thus, military initiatives are beginning to sharpen their focus on military spouses, as there is evidence that investing in their physical and mental health could have a direct effect on the health and retention of military service personnel [4].

The military lifestyle presents unique stressors such as frequent moves, isolation from family and friends, unpredictable changes, and uncertainty about the well-being of the spouse during combat operations [5]. Thus, it is not surprising that military spouses report high levels of stress and depression, particularly during deployments [6–8]. Furthermore, the “mission first” culture of the military conditions spouses to set aside their own needs, balance multiple roles and responsibilities, and possibly lose their sense of identity as they put their own career and education goals on hold to support their spouse [9].

These factors put military spouses at increased risk for poor physical and mental health. A review of the literature on military spouse health shows research has primarily demonstrated negative effects of the military lifestyle on spouses’ mental health and the marital relationship, particularly during and after deployment [10, 11]. In addition, a small number of studies have revealed concerning trends regarding obesity in military spouses, with estimates of overweight and obesity ranging from 47 to 65% [12–15]. However, military spouses’ perceptions of and participation in the health behaviors that likely contribute to these trends (i.e., physical activity and diet) have received minimal attention in the scientific literature. Two studies by Padden and colleagues demonstrated that high levels of perceived stress are associated with decreased participation in health behaviors such as exercise, stress management, and social interaction among military spouses [15, 16]. Another study found only ~ 20% of military spouses reported engaging in regular physical activity and practicing good nutrition, and only 15% of the sample reported routinely practicing stress management [17]. However, participation in these health behaviors was significantly associated with mental health outcomes such as anxiety, depression, and well-being. Similarly, Fish found overweight/obesity was positively associated with psychological distress, but negatively associated with social support among military spouses [13]. Together, these studies provide preliminary evidence that military spouses are scarcely engaging in health behaviors, but there are substantial benefits associated with doing so.

The combination of the unique stressors military spouses endure, mounting evidence that their physical and mental health is poor, and growing recognition of their instrumental role in military families make military spouses an ideal target for health promoting interventions. However, before such interventions can be developed, a more thorough examination of the factors that impede spouses’ participation in health behaviors must be conducted. Thus, the purpose of this study was to examine the most frequently reported barriers to health behaviors among military spouses. Specifically, we examined four health behaviors with well-established relationships with physical and mental health outcomes: physical activity, diet, social connection, and stress management.

## Methods

### Design

This mixed-methods study used two primary means of data collection: surveys and focus groups. All procedures were approved by the Kansas State University Institutional Review Board (Protocol #7234). The procedures for each are described in detail below.

### Surveys

Participants were recruited online via social media channels. Links to the survey were posted on Facebook pages targeting military spouses (e.g., spouse pages for Army installations across the United States). Participants were advised that the surveys would take 15–20 min to complete, and that they would be entered into a drawing for a $50 gift card upon completion of the surveys. Any individual currently married to an active duty service person was eligible to participate. Participants provided informed consent online before initiating the surveys and completed a brief demographics measure. Surveys were completed online between September and November 2014. Twenty gift card winners were subsequently randomly selected and awarded.

The present paper presents the results from open-ended questions about prevalent barriers to health behaviors. Participants were instructed: “We would like to know what you consider to be the biggest barriers to engaging in various health behaviors. For each behavior below, please list up to 3 of your biggest barriers.” The four health behaviors were (1) engaging in regular physical activity, (2) maintaining a healthy diet, (3) connecting with other people, and (4) relaxing/managing stress. For each behavior, participants could list zero, one, two, or three barriers.

After the surveys closed, data from these open-ended questions were extracted for coding. A total of four investigators were involved in coding the barriers. A separate coding sheet was developed for each health behavior to account for any behavior-specific barriers. For each behavior, the procedure was as follows: first, one investigator read through the list of barriers and created a preliminary list of codes based on commonly observed barriers. Next, two other investigators independently coded the first 50 participants’ responses. All four investigators then met to discuss discrepancies and add/refine codes as needed. Following this meeting, the two investigators independently coded the remaining responses, then the group met again to resolve discrepancies. For analysis, frequencies of reported barriers were summed. The order in which participants entered barriers was not taken into account for the analysis; all barriers were weighted equally.

### Focus groups

To further enhance our understanding of the barriers military spouses face, a series of focus groups were conducted. At the end of the survey, participants who indicated they lived in the same city and state as the research team were asked whether they would be willing to participate in a focus group session, and were prompted to enter their contact information if they answered “yes.” A total of four focus group sessions were scheduled with these participants; attendance ranged from one to four participants (total *n* = 10). Subsequently, an email announcement was distributed on post at the local Army installation asking for volunteers to participate in a focus group about life as a military spouse. Two additional sessions were conducted with individuals who responded to these announcements; one session had five participants and the other had seven participants (total *n* = 12). Focus groups were conducted between April and November 2015.

Participants provided written informed consent and completed a brief demographics survey at the beginning of the focus group session they attended. With the exception of the session that only one participant attended (an individual interview that lasted for 26 min), all other sessions were approximately 90 min in duration. All sessions had two facilitators and followed a semi-structured interview guide (Table 1). The questions did not focus exclusively on barriers, but were designed to obtain a broad understanding of life as a military spouse, and specifically how being a military spouse impacts one’s physical and mental health and participation in health behaviors, either positively or negatively. All focus group sessions were audio recorded and transcribed verbatim. Each participant received a $30 gift card upon completion of the session.

**Table 1:** Focus group semi-structured interview guide

Grand tour questions • What is it like to be a military spouse? • How does being a military spouse impact your physical health? Your mental health?	
Health behavior barriers and facilitators • Physical Activity: What about being a military spouse makes it harder to be physically active? What makes it easier? • Nutrition: What about being a military spouse makes it harder to eat well? What makes it easier? • Social: What about being a military spouse makes it harder to connect with other people? What makes it easier?	
Closing • Is there anything else you would like us to know that would help us better understand your experience as a military spouse and how it impacts your health?	

The entire data set was coded using thematic data analysis. The lead investigator read through and highlighted the transcripts deductively using the potential categories identified in the survey. The coding frame included barriers specific to physical activity, diet, social connection, or stress management. The lead investigator then sorted the codes by category, inductively identified emerging themes within the categories, and quantitatively counted the frequency of themes. Frequency was established through coding each highlighted barrier based on the list and tallying the number of times each barrier was mentioned. Exemplary quotes for the most common themes were subsequently extracted. The data were triangulated using multiple researchers. A second investigator read through all transcripts and checked the labeling, categorizing, and identified themes. The investigators discussed the results and reached consensus. Additionally, an expert and community insider reviewed the data. This third investigator found the reported results to be consistent with her experiences as a military spouse. The results were communicated through narrative reflecting the most commonly discussed barriers.

## Results

### Survey results

A total of 230 individuals provided data on at least one barrier, 228 of whom were female. On average, participants were 32.9 years old and had been married for 8.5 years (range: 1–34 years). Approximately two thirds of the sample (68.7%) had at least one child, and 29.1% of participants were working full-time. A majority of participants’ spouses served in the Army (61.3%), followed by the Navy, Air Force, and Marines. Just over half of participants were married to enlisted soldiers (55.1%); the remainder were officers’ spouses.

Table 2 reports the total number of barriers reported for each behavior, as well as the initial inter-rater reliability. Overall, there was high agreement between investigators (~ 80–90%). After discussion all discrepancies were resolved for 100% agreement. The top five most commonly reported barriers to engaging in regular physical activity, maintaining a healthy diet, connecting with other people, and relaxing/managing stress are presented in Table 3. Lack of time was the most prevalent barrier for physical activity, social connection, and stress management, and the second most prevalent barrier for diet. Financial concerns were the most prevalent barrier to maintaining a healthy diet. Barriers related to parent/family responsibilities were commonly reported across all health behaviors. Individual-level barriers such as lack of motivation, lack of self-control, and feelings of anxiety and worry also appeared in the top five for physical activity, diet, and stress management, respectively. Issues that are likely specific to the military lifestyle (i.e., moving frequently and living far from family and friends) were prevalent barriers to social connection.

**Table 2:** Number of barriers reported on surveys

	# of participants that reported at least one barrier	Total # of barriers reported	IRR
Physical activity	230	608	88.8%
Diet	223	562	81.3%
Social connection	212	513	79.5%
Stress management	206	476	80.0%

*IRR* Inter-rater reliability

**Table 3:** Most frequently reported barriers reported on surveys [n(%)]

Rank	Physical activity (*n* = 230)	Diet (*n* = 223)	Social connection (*n* = 212)	Stress management (*n* = 206)
1	Lack of time/ Busy schedule *n* = 142 (61.7%)	Financial concerns *n* = 93 (41.7%)	Lack of time/ Busy schedule *n* = 98 (46.2%)	Lack of time/Busy schedule *n* = 108 (52.4%)
2	Parenting demands *n* = 74 (32.2%)	Lack of time/Busy schedule *n* = 65 (29.1%)	Geographic distance from family/friends *n* = 37 (17.5%)	Parenting/family demands *n* = 79 (38.3%)
3	Lack of motivation *n* = 70 (30.4%)	Cravings for unhealthy foods/lack of self-control *n* = 46 (20.6%)	Transient lifestyle (frequent moves) *n* = 35 (16.5%)	Work *n* = 33 (16.0%)
4	Tired/ lack of sleep *n* = 64 (27.8%)	Time to cook/ prepare healthy food *n* = 38 (17.0%)	Parenting demands *n* = 29 (13.7%)	Feeling anxious/overwhelmed/worried *n* = 25 (12.1%)
5	Other priorities *n* = 37 (16.1%)	Kids/family prefer unhealthy foods *n* = 34 (15.2%)	Dissimilar interests *n* = 28 (13.2%)	Financial concerns *n* = 20 (9.7%)

### Focus group results

In total, 22 female spouses participated in a focus group session. On average, participants were 32.8 years old and had been married for 8.7 years (range: 6 months-25 years). Nine participants (40.9%) had no children; the remainder were parents. Three participants (13.6%) were employed full-time; the remainder were working part-time, students, or full-time homemakers. All focus group participants were Army spouses, of which 50% were married to enlisted soldiers and 50% were married to officers.

Overall, the results from the focus groups complemented and enhanced the results from the surveys. Table 4 presents the five most common barriers reported during the focus groups for each of the four health behaviors. Because the conversations during the focus group sessions focused specifically on how life as a military spouse might uniquely impact health, the most commonly reported barriers reflect this; many are very specific to the military lifestyle. Surprisingly, lack of time was not frequently mentioned directly during the focus groups, though time constraints were likely an underlying issue for some of the commonly reported barriers (e.g., inconvenience of going to the gym, effort required to build and maintain friendships). Throughout the focus group sessions participants reported putting their own health and well-being on the backburner. They discussed having too many other things on their plate, so that health behaviors like physical activity, healthy eating, and social connection did not feel like a priority. Specific barriers for each of the health behaviors are discussed in more detail below.

**Table 4:** Most frequently reported barriers reported in focus groups, with exemplar quotes

Behavior	Barrier	Quotation
Physical activity	Exercising on post is inconvenient or uncomfortable *n* = 16	*I’m not going to take 20 min out of my day to drive up on post and then workout with a whole bunch of grunts. It’s just weird to me. You’re the only female and it feels kind of weird.*
	Parenting demands/ lack of childcare *n* = 11	*Being stationed here, it’s been very difficult for me to work out because I can’t go to a gym or can’t find any with childcare. And it wasn’t that way where we came from. And I’m very picky about who I leave her with, so I don’t have a lot of sitters in the area that I could use for that.*
	Gym is only viable workout option *n* = 11	*Sometimes I think we feel a lot of pressure that we have to go to the gym to be legitimately doing something for our health, and then it’s frustrating because we think okay, well I don’t have 2 h to go to the gym, so I’m just going to sit here.*
	Have to start over with every move *n* = 7	*Once you finally get in that rhythm, you move and finding a new gym and finding people to work out with and trying to get back into that rhythm, it can take awhile and sometimes it can take so long that your new rhythm is not exercising.*
	Lack of motivation *n* = 7	*I have no interest in exercising whatsoever. Zero. None. It just seems like something that would be such a pain to me, that I just have no desire to do it.*
Diet	Expensive to eat healthy foods *n* = 11	*It’s almost like, you should eat healthy but you can’t afford to. And I think that’s a huge thing for military wives, cause we don’t make a lot. We have a set amount for food each paycheck, so for us, we don’t eat as healthy as we should because we cannot afford it. It’s just so expensive*.
	Don’t like to cook for one *n* = 9	*It is definitely a struggle eating for one. I am not a cook to begin with so to be honest, during deployment, 50% of my meals were cereal. Because when I got hungry I wanted to eat. And I didn’t want to make something, and the amount of money it takes to make a full meal for one person is horrible.*
	Spouse/family want to eat junk *n* = 8	*If I want to eat something that’s better for me, my husband doesn’t like the same food that I do, so I forget what I want to eat cause I want to feed him. So I will make him lasagna even if I want to just have a salad*.
	Fast food is easier and more convenient *n* = 8	*It’s so much easier to go through the drive-thru and grab something on the way home, even if you have stuff to cook, you have the money, it’s just easier sometimes, because you don’t get home until 7 or 8 o’clock at night, and then you know you don’t want to cook a full meal.*
	Lack of knowledge about healthy foods *n* = 5	*I wonder with all the 18 and up wives, if they even know how to cook and prepare things for themselves. Who knows what they learned at home and went right into married life.*
Social connection	Too much effort to make plans or develop friendships *n* = 11	*It’s almost work to try to find friends when you are a military spouse. Cause not only do you move around so much, but you just differ in so many ways, because everyone has their different places, and everyone has different values or interests. So I would say it’s more work than anything.*
	Spouse groups are cliquey or catty *n* = 10	*The FRG can sometimes be a very…you know…not always a positive thing. A lot of people don’t join them on purpose because they can be really catty and gossipy.*
	Spouse’s rank affects social interactions *n* = 7	*You deal with way more stereotypes and being stigmatized. You know my husband’s enlisted, but I went to college…does anyone ever ask me about my experience? It’s just what is your husband, who is your husband… no I’m a person separate from him and would like to talk about what I do. It just always felt like first and foremost let’s just make sure we put the enlisted in their place.*
	Difficulty relating to others from different backgrounds *n* = 7	*When we moved in Texas, each neighborhood we went to people were really nice and welcoming, and then we got here, and we are on post, and it’s like nothing. I was like WOW. So I haven’t put myself out there, and it’s only been in the last 6 months or so where I have made an effort to go out there and meet people.*
	Difficulty relating to others with or without kids *n* = 7	*I would say it’s hard for me to make friends because I don’t have kids and I know that others do. Try being 34 and then trying to find another 34-year-old couple who doesn’t have kids. It’s very hard.*
Stress management	Feel insignificant/ not valued *n* = 8	*I think that’s also a hard part of being a military spouse is the Army is first, your husband is first, his career is first, and you come second. So saying ‘I am worth it’ to find and do stuff for you.*
	Reluctant to ask others for help *n* = 6	*You have to rely on strangers at one point, and when somebody is asking you if you need any help, you just need to stop saying no. Taking care of ourselves, you know if we don’t take care of ourselves, nobody else is going to get taken care of.*
	Deployment/ uncertainty about spouse’s well-being *n* = 6	*You keep yourself going going going so that you don’t think too much about whatever situation.*
	Overwhelmed by doing it all on her own *n* = 5	*I was literally [getting sick] and trying to give my son a bath at the same time. I know that’s terrible but I mean, it is stressful, and it is a lot of hard work, I think especially when you have kids, because you have to play both roles.*
	Uncertainty/ inability to plan *n* = 5	*Things are changing all the time. It’s so hard to plan your life, knowing in the back of your mind he might not actually be around to do it.*

#### Physical activity

During the focus group sessions, discussions about physical activity focused almost exclusively on exercising in a gym setting. Participants explained that although they can access the gyms on post for free, they rarely use them because they are inconveniently located, do not offer childcare, and are used primarily by male soldiers. For individuals with children, their childcare options were to use a daycare center on post that was located 15–20 min away from the gym, or to bring their children along and attempt to supervise them and keep them occupied while working out. Both options were highly unsatisfactory and made using the gym impractical for parents. For individuals without children, using the gym was still unappealing because they felt uncomfortable in a setting designed for and mostly used by male soldiers. As the only female in the gym, participants felt like they were “on display,” and some also described discourteous behaviors by soldiers including leaving heavy weights on the equipment, not wiping down equipment after use, and “hogging” equipment during peak times. Because of these issues, some participants described pursuing gym memberships elsewhere, but for many this was cost prohibitive, and still did not address the issues of lack of time and lack of childcare.

The perception that one has to go to a gym to exercise may have been exacerbated by the fact that participants were military spouses. Although they acknowledged that walking and other daily activities can “count” as physical activity, they felt the military culture made them especially resistant to accepting that mindset. Because the military focuses on intense physical training, this was how participants defined exercise. Unfortunately, having such a rigid view of exercise often led individuals to avoid physical activity completely. They felt that if they could not set aside 1 or 2 h for vigorous exercise, the alternative was to do nothing at all. Of course, these barriers were sometimes compounded by a lack of motivation. Some participants admitted that they simply did not make exercise a priority, perhaps because they found it to be unpleasant or because other stressors were more pressing. Many participants viewed exercise as an added chore amidst an already demanding lifestyle, and when they craved a break from those demands, they were more likely to cope with food or, in some cases, alcohol.

Some participants also discussed challenges associated with the necessity to frequently move and “start over.” They described getting into a consistent routine at one location (e.g., finding a gym they liked, an exercise group or partner they liked, etc.) and then having difficulty re-establishing a routine at a new location. These issues were exacerbated for individuals with children, as they had to explore new daycare options as well. In addition to the logistics of researching gyms and other exercise resources, participants described an added mental load that was, at times, paralyzing. They were so tired of having their routines and lifestyles interrupted that during times of transition they focused on the necessities (housing, school, work, etc.) and had limited energy remaining to worry about “extras” such as exercise.

#### Diet

In line with the survey results, the expense associated with eating healthy foods was the most commonly reported barrier to healthy eating mentioned during the focus groups. Many participants explained that although they would like to eat healthfully, they tended to opt for packaged or processed foods that they perceived to be more affordable than fresh produce and lean meats. This was especially true among spouses of enlisted soldiers, whose fixed income was limited. However, others argued that financial barriers are more of a “budgeting problem” that can be addressed by educating individuals about how to allocate enough money for food and eat well on a budget. Similarly, although lack of knowledge about healthy foods was mentioned during most focus group sessions, it was primarily in regards to participants’ perceptions of barriers *other* spouses faced. Most individuals believed they themselves had sufficient knowledge about nutrition, but perceived other spouses (e.g., young lower enlisted spouses) were uneducated about how to purchase and prepare healthy foods.

In spite of their knowledge of what constitutes a nutritious meal, participants believed their military lifestyle made it difficult to maintain a healthy diet. The frequent absence of their spouses, either due to deployment or long work hours, presented obstacles for participants both with and without children. Participants without children reported low motivation to “cook for one” due to the time and money required to prepare a full meal. At times feelings of depression and loneliness due to their spouse’s absence suppressed their appetites and further exacerbated these motivational struggles. While some participants with children reported that they made a greater effort to cook and provide balanced meals for their children, others reported similar motivational barriers. They knew their children would be content with processed foods or fast foods, so they did not invest the energy to cook anything that would require more effort. Overall, the combination of the time and motivation required to prepare healthy meals led individuals to gravitate towards more quick and convenient options, including processed foods at home and fast food on the go.

On the other hand, other participants found maintaining a healthy diet to be more difficult when their spouses *were* home. They said their husbands preferred unhealthy foods, so they would eat what their husbands wanted, even if it wasn’t their personal preference. Several participants reported their spouse’s desire for junk food peaked immediately following a deployment or extended separation. After having limited access to favorite foods or restaurants for months, “all he wants to eat is crap.” In general, it was clear that participants felt their eating patterns were significantly impacted by the frequent “cycles” associated with military life, including deployments and changes in duty station.

#### Social connection

Barriers to social connection mentioned in the surveys and during the focus group sessions were generally similar. Lack of time was the most common barrier reported on the surveys, and the focus groups provided additional context to explain why the military lifestyle often prevents individuals from investing time in friendships. Specifically, spouses viewed the process of meeting others and making friends as “work” that was not worth the time and effort it involved. Most military families move every 2 to 3 years, so taking the time to genuinely get to know other spouses felt fruitless with the knowledge that one would have to move on and start over again soon. Whereas this led some participants to withdraw from social interactions completely, others found acquaintances with whom they could spend time but avoided forming “deep friendships” to protect themselves from the pain of disconnecting. Another factor that contributed to time-related barriers was that participants were often trying to “do it all” on their own while their husbands were away. Thus, as with exercise, they viewed social connection as something extra that they did not have the time or energy to prioritize. They felt overwhelmed themselves, and assumed other spouses felt the same way, which prevented them from reaching out to pursue friendships, or ask others for help.

Many participants reported that being a military spouse made it difficult to relate to others. Because they were frequently moving, they were often surrounded by individuals with different values, backgrounds, and life experiences. Some participants found certain duty stations to be “friendlier” than others, and felt especially isolated when they moved to a new post and no one reached out to welcome them. Some participants also discussed challenges forming or maintaining relationships with civilian friends, due to difficulty understanding and empathizing with the other’s lifestyle. They explained that although civilian friends tried to empathize with their situation, it became clear that they didn’t *really* understand when they made comments comparing their husband’s business trip to deployment, or offered to help but then didn’t follow through. Additionally, participants without children often found it difficult to connect with spouses with children, and vice versa. Individuals with children found their lives revolved around their children’s schedules and activities, so they were unavailable for spontaneous dinners or happy hours with friends, whereas individuals without children felt most social activities were geared towards families, and they didn’t fit in.

One specific barrier to social connection that was raised during most of the focus group sessions was the issue of partner’s rank. By and large, participants felt that the hierarchical “class system” created by the military made it difficult to cross boundaries and form friendships. Although some participants justified this dichotomy by explaining that it is difficult to become overly chummy with the spouses of soldiers their husbands supervise, others felt it was unnecessarily contentious. Many spouses of enlisted soldiers felt they were “looked down upon” by officers’ wives, simply because of their husband’s rank. On the other hand, some officers’ wives felt stereotyped as well. They believed officers’ wives were usually portrayed as haughty and pretentious, and found it difficult to transcend those labels. Outside of rank, many participants believed military spouses are “cliquey” or “catty” in general. Although Family Readiness Groups (FRGs) are designed to provide a support system for military spouses, many participants had had negative experiences with them, and thus chose to avoid them as a potential avenue for meeting friends.

#### Stress management/mental health

Difficulties managing stress and mental health were discussed frequently during the focus group sessions. Often the stress was related to the need for military spouses to manage everything unassisted. Participants commonly discussed challenges associated with balancing work, childcare, and household needs alone while their spouses were deployed or on extended separations. Even when their spouses were not away, participants described long and unpredictable work hours that required them to be available for childcare and other responsibilities at all times. In addition to the logistics of handling everything alone, participants felt there was added pressure from the perception that military spouses are *supposed to* take care of everyone else. They described a culture where the soldier and his career come first, and the needs of the spouse and family are a much lesser priority. Because of these perceptions, participants were often reluctant to seek or accept help from others, even when they needed it. However, some acknowledged that letting go of those apprehensions and asking for help was not a sign of incompetence, but rather an essential step for maintaining one’s mental health and carving out some time for self-care.

A major stressor that was often discussed was the uncertainty associated with military life. Deployments were certainly a time of high stress, during which spouses reported high levels of anxiety regarding their spouse’s well-being, as well as depression triggered by loneliness and, at times, strained or limited communication with their spouse. The uncertainty went beyond deployments and separations, however. Participants reported that plans and schedules changed often, and as a result their plans were frequently “derailed completely and utterly.” On a day-to-day basis, they could be expecting their spouse home at 5 or 6 p.m., but then an emergency arises and they get home at 9 or 10 p.m. instead. Others discussed having vacations or special events planned, but then finding out days before that their spouse has to leave town for a week. For individuals that preferred to have a set schedule and plan in advance, the military lifestyle was clearly incompatible with this preference. Overall, this uncertainty contributed to an underlying sense of stress that never truly abated. Participants described being constantly “on edge” knowing that things could change at any second, and not being able to enjoy the sense of relief others might experience when stressful situations have a finite end.

## Discussion

This study was designed to examine the most prominent barriers to health behaviors among military spouses^1^. Although previous research has examined a variety of mental health outcomes, and the factors that contribute to them in this population, research on participation in health behaviors among military spouses is scarce. Our findings suggest military spouses do face numerous barriers to engaging in physical activity, maintaining a healthy diet, managing stress, and connecting with others. Some of these barriers are similar to those commonly reported by civilians, while others are more directly related to the military lifestyle. In general, participants in our study did not view participation in health behaviors as a priority, and reported that there were often more pressing concerns that prevented them from investing time in self-care.

The pressure to “do it all” and put others’ needs first is not unique to military spouses and has been identified as a barrier that constrains leisure activities for many women [18]. However, several aspects of military life may further exacerbate these perceptions: a) military culture views spouses as dependents whose primary purpose is to serve their husbands in support of the military mission, b) the active duty spouse is so frequently absent that his partner is left to shoulder all of the burdens alone, and c) military families frequently live far from family and friends, and have no support system in place [9, 19, 20]. Thus, for military spouses, doing it all takes on a more literal meaning and is not merely a function of her perception of her role as a woman or a spouse. Despite the benefits of support from others, many spouses in our sample were reluctant or unwilling to seek support for fear of being perceived as a burden or incapable of coping with life’s demands. Similar feelings of reluctance to seek help for mental health problems have been reported in previous research [10, 11]. Furthermore, the desire to appear “in control” may be exacerbated by the fact that compared to civilian spouses, military spouses may feel a more pervasive loss of autonomy because so many life circumstances are beyond their control, and instead are a function of their spouse’s career [19, 21].

Based on the themes that emerged from the focus group sessions, the transient nature of military life appeared to contribute to barriers for all four of the health behaviors examined. Most military families undergo a change in duty station every 2 to 3 years, and these frequent relocations have been shown to negatively impact employment and education outcomes and opportunities [20, 22], access to healthcare [22], and children’s education, social relationships, and psychological well-being [23]. The present study adds to this literature by suggesting that these moves were not only an ongoing source of stress and uncertainty for military spouses, but they also made it difficult for them to maintain diet and exercise patterns, and to build and maintain meaningful relationships with friends. These findings are similar to another qualitative study of military spouses’ perceptions of leisure, in which participants reported relocations had a significant impact on their leisure activities, and that they had to make significant sacrifices every time they moved [9]. Because of the heavy physical and emotional toll these frequent moves played on spouses and children, they were one of the primary factors contributing to spouses’ perceptions that the military does not value family. Unfortunately, perceptions of inadequate concern and support for military families have often been reported by military spouses in previous studies [19, 23, 24].

The impact of these frequent moves on spouses’ support networks was particularly pronounced. Participants valued and craved social interaction with others, but also noted multiple challenges associated with building and maintaining friendships. For military spouses, investing the effort to make friends can feel fruitless when they know they will be moving and starting over again within a few years. Other studies have reported similar findings, with spouses characterizing making friends as “a real uphill slog just to meet similar folks” [19] and commenting “People get so tired of starting over all the time so after awhile you just don’t bother” [9]. Compounding these challenges, the issue of military rank adds another layer to social barriers for military spouses. It was apparent from the focus group sessions that spouses of officers and spouses of enlisted soldiers rarely comingle, and additional research is needed to understand the extent to which these divisions are “necessary” based on the military hierarchy, or whether tensions could be alleviated by interventions that address stereotypes and strategies for transcending them. Overall, these pervasive social barriers are concerning considering research has shown that social support from friends contributes to heightened psychological well-being among military spouses [25] and can buffer the effects of life stressors on spouses’ depression [4]. As military spouses are frequently stationed far from their families of origin, establishing a “sense of community” wherever they are can fulfill needs for connection and belonging [26]. Military installations should continue to explore spouses’ preferences for support networks and provide resources for connecting those with similar interests. Of course, it is also incumbent upon the spouses to seek out and use such resources.

Although some spouses did not view physical activity and healthy eating as priorities, almost all participants were concerned about their mental health, perhaps because they could see the immediate adverse effects of stress, depression, and anxiety on their quality of life and the quality of life of their spouses and children. Previous research has demonstrated that military spouses do face elevated levels of stress and depression, and that aspects of military life such as deployments and extended separations can have a significant negative impact on spouses’ mental health [7, 24, 27]. Furthermore, there is evidence that poor mental health (e.g., anxiety) can negatively impact spouses’ perceptions of their overall health [28]. These results suggest that future health-promoting interventions might be better received if they are framed as holistic health programs to enhance mental health and quality of life. This strategy is likely to facilitate autonomous motivation among participants because health behaviors such as physical activity and diet are linked to outcomes they truly value, as opposed to extrinsic outcomes (e.g., weight loss) that take a long time to achieve (if they are achieved at all) and contribute to perceptions that one “should” be exercising and/or dieting [29]. In addition, to directly address mental health, interventions can incorporate strategies such as problem-focused coping, which empower individuals to take constructive steps to cope with stressors, and have been shown to alleviate physical symptoms of stress even when perceptions of control are low [6, 30].

In terms of physical activity, the focus group sessions shed light on the factors that might contribute to the frequently referenced barrier of lack of time. Spouses tended to have a narrow definition of what counts as exercise and think almost exclusively about exercising in a gym setting. These perceptions could also underlie other frequently reported barriers such as lack of childcare and lack of motivation. If individuals do not perceive exercising in a gym setting to be convenient or enjoyable, this will likely curtail their desire to participate in physical activity, and perhaps increase their preference for sedentary leisure pursuits [31]. Participants in this study characterized gyms on post as inconvenient and unsuitable for spouses, which is backed by previous research demonstrating spouses are considerably less satisfied with military fitness facilities than service personnel [32]. These results suggests future interventions should consider shifting their focus away from promoting exercising in a gym, and instead emphasize other types of physical activity that could more easily fit into individuals’ existing lifestyles. These alternative types of physical activity would not only be more feasible, but also more enjoyable. For example, walking is a simple activity that can be easily integrated into one’s daily life, and if framed as a means to enhance quality of life, can conjure feelings of pleasure as opposed to feelings of dread often associated with prescribed vigorous exercise in a gym setting [33]. Considering the many roles and responsibilities military spouses must balance, framing physical activity as something that can be done in short bouts may help alleviate barriers related to limited time, motivation, and childcare.

The expense associated with eating healthy was the most commonly cited barrier from both the surveys and the focus groups. To our knowledge, only one other study has specifically examined barriers to healthy eating among military spouses, and similarly found barriers related to the time and expense associated with following a healthy diet were associated with increased body mass index in this population [14]. Other studies in the general populations of the US, Australia, and Switzerland also support these findings. A qualitative study of barriers and facilitators to dietary guidelines adherence across six states in the US found that core barriers to healthy eating for adults included taste, difficulty of changing habits, cost, and lack of nutrition knowledge, cooking skills, and recipes [34]. Another study in Melbourne, Australia examined barriers among women of varying socioeconomic status and found being less health conscious, having a lack of time due to family commitments, and perceiving a high cost to healthy eating were the most commonly reported impediments to maintaining a healthy diet [35]. Finally, a study in Switzerland investigated trends in prevalence of barriers to healthy eating, and found price was reported as a barrier by 39–52% of the population over the 15-year survey period [36]. Based on these results, future interventions designed to improve dietary behaviors among military spouses should consider evaluating the reality of the perceived high cost of healthy eating on military bases, providing resources to support healthy eating on a budget, and making healthy food items more affordable and accessible to all military spouses and families. Additionally, educational content can address financial barriers by teaching individuals to plan ahead, to cook enough to have leftovers, to buy more store brands, to sign up for and read sales flyers, to buy in bulk and split items with a neighbor, to replace canned beans for meat, and to involve friends in meal preparation/ sharing events [37].

The results of this study should be considered in the context of its limitations. First, it is important to acknowledge that these findings may not reflect the experiences of all military spouses. A majority of participants were female Army spouses, and all voluntarily participated. Advertisements for the surveys specifically included the word “health,” and thus may have appealed to individuals interested in health and wellness. However, participants still reported a large number of barriers to the health behaviors assessed. Additionally, both samples included spouses who had been married for varying lengths of time ranging from a few months to 25+ years, and both had a roughly equal distribution of spouses of enlisted soldiers and officers. Thus, the perspectives and experiences of a wide range of spouses were included. Second, this study only focused on four specific health behaviors. Although there is good evidence that engaging in these behaviors will have a positive impact on physical and mental health, other health behaviors (e.g., seeking healthcare, limiting substance use) may be equally important, and warrant attention in future studies. Third, this study only focused on barriers, and not on facilitators or factors that may help alleviate those barriers. Although most of the focus group conversations primarily focused on challenges associated with being a military spouse, some participants did identify benefits of being a military spouse such as building resilience, forming support networks, and being exposed to new experiences. Future research should further explore these facilitators, and ways to leverage them to positively impact spouses’ health. Finally, this study only included military spouses as participants, so the extent to which the barriers reported are unique to, or more prevalent among military spouses warrants further investigation. Future studies should consider comparing barriers between military and civilian spouses to better understand which factors are unique to the military lifestyle.

## Conclusions

Military spouses are an important, but understudied population that exhibits poor physical and mental health compared to the general population. This study was one of the first to thoroughly examine the unique challenges and life situations that may impact the health of military spouses and their families. The results of this study clearly demonstrate that military spouses face unique barriers to engaging in health-promoting behaviors, and are an ideal target for health behavior interventions. Although some of the common barriers reported are a function of aspects of military life that are outside of individuals’ control (e.g., moving frequently), others could be addressed in interventions by reframing individuals’ perceptions of health behaviors and teaching strategies for coping with challenging but predictable obstacles. Because military spouses’ health is intimately tied to the health of their families, developing initiatives that focus on improving spouses’ health is an important public health priority.

## Footnotes

1 Because the military spouses in the present sample were almost exclusively females married to males, throughout the discussion we will refer to military personnel as males and spouses as females. We acknowledge that these roles are not always divided in this way, but our data do not speak to the unique issues that male military spouses might experience.
